# Ion Complexation Explains Orders of Magnitude Changes in the Equilibrium Constant of Biochemical Reactions in Buffers Crowded by Nonionic Compounds

**DOI:** 10.1021/acs.jpclett.1c03596

**Published:** 2021-12-28

**Authors:** Krzysztof Bielec, Adam Kowalski, Grzegorz Bubak, Emilia Witkowska Nery, Robert Hołyst

**Affiliations:** † Institute of Physical Chemistry, Polish Academy of Sciences, 01-224 Warsaw, Poland; ‡ Institute of Chemical Sciences and Engineering, EPFL CH C2 425, Bâtiment CH, Station 6, Lausanne CH-1015, Switzerland

## Abstract

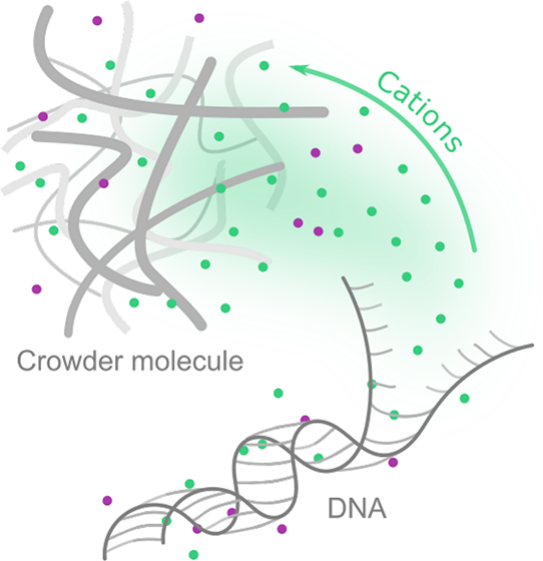

The equilibrium constant (*K*) of biochemical complex formation in aqueous buffers with high concentration (>20 wt %) of nonionic compounds can vary by orders of magnitude in comparison with the *K* in a pure buffer. The precise molecular mechanisms of these profound changes are not known. Herein, we show up to a 1000-fold decrease of the *K* value of DNA hybridization (at nM concentration) in standard molecular crowder systems such as PEG, dextrans, Ficoll, and glycerol. The effect responsible for the decrease of *K* is the complexation of positively charged ions from a buffer by nonionic polymers/small molecules. We determined the average equilibrium constant for the complexation of ions per monomer (∼0.49 M^–1^). We retrieve *K*’s original value for a pure buffer if we properly increase the ionic strength of the buffer crowded by the polymers, compensating for the loss of complexed ions.

Biochemical reactions occur in the cytoplasm of living cells crowded by biomolecules. They occupy up to 40 wt % of the cell interior.^1,2^ The solutions of nonionic compounds at large concentrations (∼40–50 wt %) are *in vitro* models of the cell’s cytoplasm. In these solutions, the equilibrium constant of biochemical reactions often decreases by orders of magnitude compared to pure buffers.^3−6^ The mechanism of this phenomenon is not known. Herein, we show up to a 1000-fold decrease of the *K* value of DNA hybridization (at nanomolar concentration) in standard molecular crowder systems, such as PEG, dextrans, Ficoll, and glycerol. We prove that the general mechanism responsible for decreasing *K* is the complexation of positively charged ions from a buffer by nonionic polymers/small molecules. We confirm the mechanism by restoring *K*’s original value for a pure buffer by adding a calculated amount of ions to the solution to compensate for the loss of complexed ions. Typically, the buffer’s ionic strength is ∼100 mM, and nonionic compounds often reach ∼1 M concentration. Therefore, even a very weak complexation of ions, say at ∼1 M^–1^, removes half of all ions from the solution and decrease the equilibrium constant.

The molecular crowding phenomenon is also studied because a high concentration of cosolutes can alter molecules’ structures, diffusion coefficients, and binding rates.^7−11^ The crowded environment is frequently recreated artificially to understand biochemical reactions occurring inside living cells.^12,13^ Cosolutes used in solutions mimicking cells’ interior have to be chemically inactive. It was shown that cosolutes can increase the *K* at modest crowder concentrations (∼10 wt %) through one of the crowding mechanisms—depletion forces.^3,14^ The origin of those forces is caused by a decrease of the local volume around the biomolecule by crowders, increasing the effective concentration of reactants. Such a mechanism was shown in the example of the formation of DNA hairpins and the reactive structure of proteins/enzymes.^15−17^

The majority of biochemical reactions (*e.g.*, DNA hybridization or protein folding) require well-defined thermodynamic conditions, including pH and ionic strength (IS). Thus, the experiments in crowded systems are frequently performed in fixed buffers at the physiological IS of around 100 mM. However, the living cell’s interior is an active, self-regulating system that can control ion concentration (*e.g.*, through the ionophoric transfer of cations). The most popular crowding polymer, PEG, complexes divalent cations in nonaqueous solutions with the equilibrium association constant below 1 M^–1^.^20−23^ This raises a question: Can crowders being in high concentration (*i.e.*, 10–50 wt %) affect the IS of the standard buffers (*e.g.*, phosphate buffer) and, in consequence, change *K* of the complex formations? In aqueous solutions, the formation of complexes between nonionic polymers and cations is difficult to observe directly by experiments because of the strong hydration of metal cations.^24^ However, some studies explained interactions of PEG moieties with nonionic and anionic surfactants in water-based system as mediated by alkali metal cations, therefore revealing PEG–cation complexation.^25,26^ Moreover, Ohki *et al*. by cooperative application of ion-transfer voltammetry at a liquid/liquid interface together with X-ray absorption fine structure (XAFS) measurements strongly suggested that alkali cations form a complex with PEG in water even in relatively low PEG concentrations (5–16 wt %).^24^ In addition, Breton *et al*. confirmed complexation of (Na^+^, K^+^, Rb^+^, and Cs^+^) indirectly in the electrophysiology experiments by analyzing partitioning of neutral, flexible PEG 2000 molecules into the α-HL nanopore. In the presence of the mentioned cations, the neutral polymer behaved as if it was charged. Interestingly, for lithium cations (Li^+^) that effect was not observed.^27^ The formation of PEG–sodium complexes was also used in cation template-assisted cyclopolymerization.^28^

In this Letter, we revise molecular crowding’s effect on biochemical reactions at the nanomolar concentration range using a previously validated brightness-based method.^18,19^Figure 1 presents the schematic concept workflow. We studied DNA hybridization in standard molecular crowder systems (*i.e.*, PEG, EG, glycerol, Ficoll, and dextrans) as a noncovalent complex formation model. The DNA hybridization is ionic strength-sensitive and is discouraged in a low ion concentration environment.^29^ Thus, it is a good indicator of ions’ potential binding by crowders. We also determined the relationship between *K* and IS to support our hypothesis. In addition, we confirm binding of sodium by dextran in aqueous solution by independent potentiometric measurements using an ion-selective electrode. Finally, we combined both approaches to show that not the mere physical abundance of crowders, which changes the volume available to reactants, but the complexation of ions by them is responsible for changing the *K* of reaction.^20−23^

**Figure 1:**
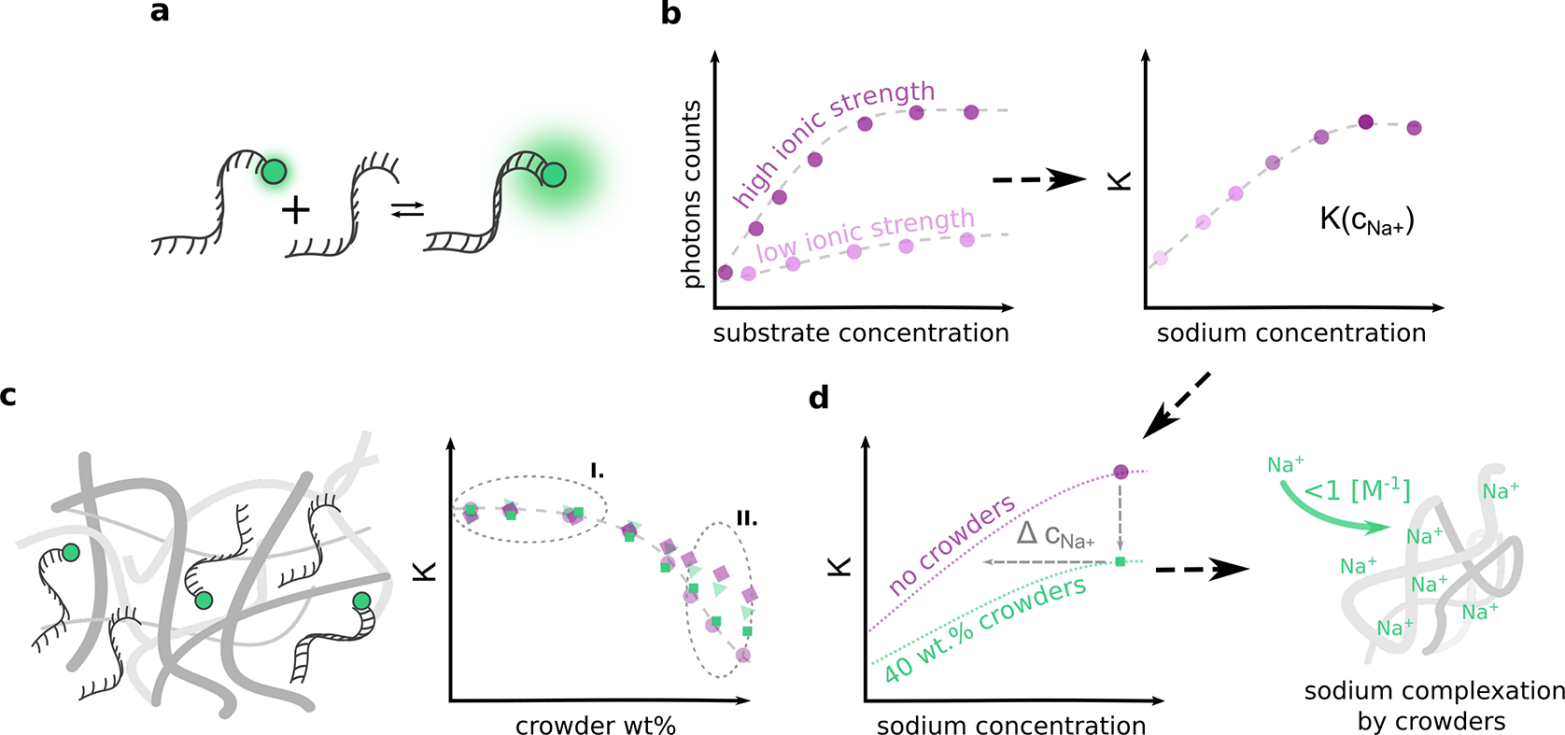
Hybridization of complementary DNA oligonucleotides was used as a model biochemical reaction. (a) The change of the fluorescent properties as a result of the complex formation makes it possible to use the brightness analysis method.^18,19^ We investigated two biophysical factors that affect the formation of the noncovalent complex: ionic strength and crowded environment. (b) Ionic strength. The formation of the complex was observed at different ionic strengths. (c) Crowded environment. We used the most common biorelated crowders of different sizes and chemical structures. The influence of cosolute is negligible even at 10^9^ excess over the concentration of substrates (region I). There is no correlation between sizes and structures of different agents (region II). (d) The comparison of the interactions in a crowded environment with respect to different ionic strengths allowed the determination of the sodium cation complexation by crowder molecules.

First, we checked the influence of crowders on the DNA hybridization equilibrium constant. The formation of a double-stranded DNA backbone is an electrostatic interaction between two complementary, negatively charged strands. We monitor the effect of the crowded environment on the hybridization of complementary strands in the biochemical concentration regime (5 nM). All crowders we were using are known to be chemically inert. Dextrans and ficolls are “similar” in chemical structure as they possess sugar moieties rich in hydroxyl groups, whereas EG and PEGs are alkoxyl-rich. Using the brightness analysis method (described in the Supporting Information, section S3), the effect of crowders was observed by the determination of the equilibrium constant (*K*) of DNA hybridization.

The crowding effect on the thermodynamic stability of DNA duplexes formation was previously determined in the presence of various crowders.^5,30^ Here, at low crowders concentrations (<10 wt %), the change of *K* is nearly negligible (see Figure 2). The influence of the crowded environment changes the *K* by 2–3 orders of magnitude for the high concentration of crowders (40 wt %<). In terms of the molar concentration, this value corresponds to values even above 10 M. This means that the crowders are in over 10^9^ times excess over the concentration of substrates in this reaction (∼1 nM). Hence, if the crowding effect were significant, it would be observable at much lower concentrations. Therefore, the effect responsible for changes of *K* is not secondary interaction with crowder molecule nor effect caused by depletion with it, but rather some weak effect or interaction.

**Figure 2:**
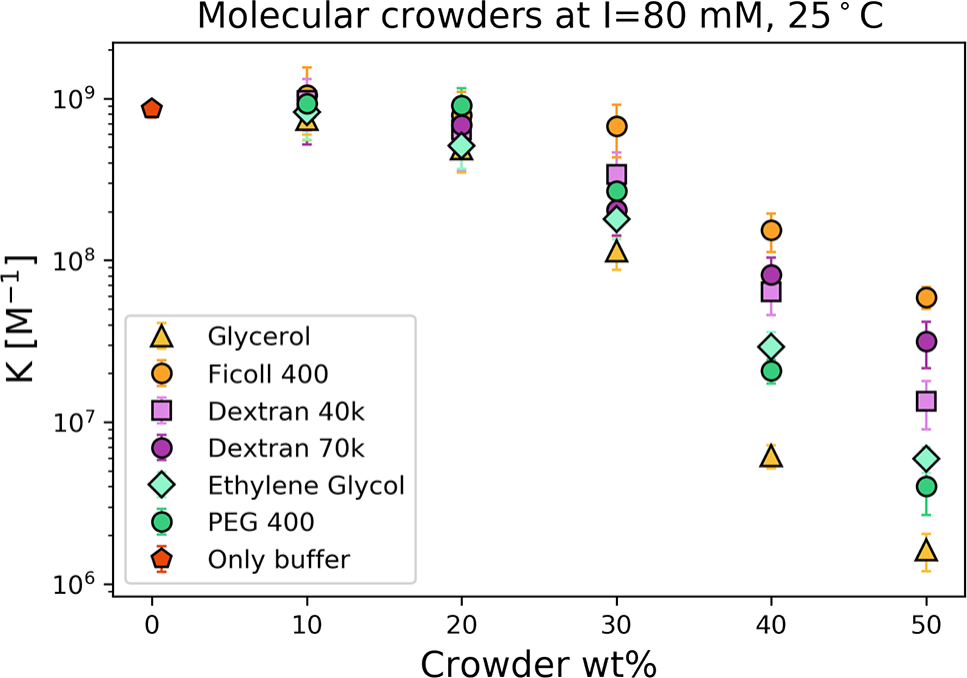
Hybridization reaction was measured at constant ionic strength in the presence of various crowder agents. The effect of crowder molecules on reaction components (presented in nanomolar concentration) is negligible below a few wt % of crowder concentration (∼200 mM). However, when the molar concentration of the crowder is approximately a few molar, the reaction is affected.

It was reported that crowder molecules, such as polyethers or carbohydrates, form complexes with cations suspended in the nonaqueous solvent.^20−22,24,31−33^ The reported values for complexation of the cations by different PEG molecules range from 1 to 10^2^ M^–1^. These values depend on the charge (*e.g.*, Na^+^ or Ca^2+^) and the type of conjugated acidic residual (strong or weak, *e.g.*, phosphate or acetate).^34,35^ If we consider a system crowded by 40 wt % PEG 400 (2.68 M) in 0.1 M PB buffer (only sodium cations) and assume no interactions due to depletion forces, then based on reported sodium complexation, κ = 1 M^–1^, the initial concentration of sodium ions drops by ∼73%.

The concentration of salts regulates the physiological processes of the cell. The changes of IS shift the equilibrium of ions of those biochemical noncovalent complexes, such as complex-forming polypeptides (antibodies), stabilization of membrane-building anionic phospholipids, protein–DNA, DNA–drug, metabolic-substrates with carboxyl or phosphate group, *etc*. Also, the activity of enzymes or receptors is controlled by the formation of ion-based complexes with specific cations to obtain proper active conformation.^36−39^

Taking all this into consideration, the decrease of the equilibrium constant in a crowded environment (shown in Figure 2) is observed because of ion deficiency caused by complexation of cations in the solution. The decrease of ion concentration in the Debye double layer reduces the screening of negative charges on oligonucleotide backbones. This results in electrostatic repulsion between DNA strands and thus the lower bound fraction. The few nanometer-sized ion-crowder complexes are too large to stabilize DNA hybridization as the average hydrogen bond between complementary nucleotides is 0.2–0.3 nm.^40^ DNA duplex formation would require such an object to detach and diffuse. In contrast, angstrom-sized ions can fill the spaces between nucleotides and their proximity without interrupting hybridization.^41^

Following that, we checked the influence of ionic strength on DNA hybridization. The effective ion concentration in the solution is described by IS, and it determines the effective electrostatic repulsion distance (Debye length). During the DNA hybridization, the base pair repulsion (caused by phosphodiester groups) is partially neutralized by cations (*e.g.*, Na^+^, K^+^, or Mg^2+^). We investigated the hybridization of 13 bp complementary DNA strands at different ionic strengths (in a range of ∼100 times lower than physiological concentrations and at a highly saline environment) (see Figure 3). We used PB buffer of various concentrations, where only sodium ions were introduced as positively charged species. At each IS, using the brightness analysis method, we determined the equilibrium constant *K*. The results were also validated by FRET (see the Supporting Information, section S4).

**Figure 3:**
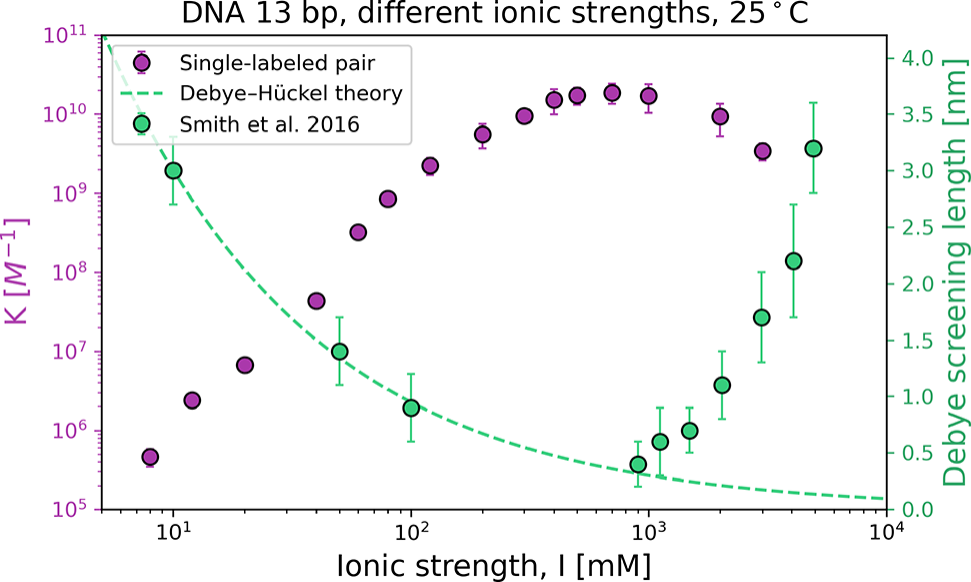
Influence of ionic strength on the hybridization of complementary DNA oligonucleotide strands (violet points). The data were compared with the observed change of Debye length recorded by Smith *et al*.^42^ The scatters represent data points, and the line (both marked in green) shows dependence of Debye length as a function of ionic strength according to Debye–Hückel theory.

The increase in salt concentration changes the binding energy between complementary DNA fragments to a significant extent. The presence of sodium cations and phosphate anions with a total IS of 10 mM generates an increase in the constant *K* by 10^6^ M^–1^ in relation to pure water, where the reaction does not take place.

The ions in the solution screen the negative charges found on the phosphate groups of DNA backbones. Accordingly, the Debye length and the effective negative charge of the oligonucleotides are reduced. Dispersive interactions start to dominate over the repulsive electrostatic forces between DNA strands. As a result, the probability of forming a DNA complex increases. For IS greater than 700 mM, the *K* constant decreases. At 2 M ionic strength, the value of *K* is 10 times lower than the maximum value observed. In this regime, the charge on the oligonucleotides is strongly screened and deviation from Debye–Hückel theory occurs.

We suggest that repulsion due to the densely packed Debye layer prevents effective collisions (interactions between DNA molecules) and hinders formation of the double-stranded DNA. A mutual effect was previously observed on the surface covered by charged nanoparticles at different ionic strengths.^43^ Moreover, Smith *et al*. using a surface force apparatus measured similar anomalous changes in Debye’s screening length at high salt (*i.e.*, NaCl) concentrations.^42^ In Figure 3, we show their data and the classical Debye screening length overlaying the *K* constant determined in our research as a function of IS. The changes in Debye length follow the inverted function of changes in *K* for DNA hybridization caused by varying IS. Following the surface-oriented Smith *et al*. experiments, we observed analogous results in bulk solution of reagents, by linking directly the influence of ions on the Debye length, and finally with the *K* of DNA hybridization.

Finally, we determined the complexation of sodium ions by crowders. The interaction scheme of ion complexation by different crowders is presented schematically in eq 1. The binding site for cation within the crowder structure may differ even between crowders of the same binding moiety (functional group). Therefore, we calculated the interaction with crowder per molecule or monomer (in the case of polymers). This model simplifies the interactions between ions and crowder molecules. Hence, the obtained values of the ion complexation equilibrium constant, κ, can be corrected by a factor dependent on the number of monomers/molecules participating in the binding site.
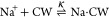
1The equilibrium constant for this interaction can be written as
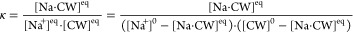
2where [Na^+^]^0^ and [CW]^0^ are initial molar concentration of sodium ions and crowder molecules, respectively.

The method of buffer preparation enables keeping the pH constant even at different ionic strengths.

To determine the ion complexation, κ, we designed an experiment with the following methodology: we always controlled the total number of sodium ions by preparing a given buffer concentration; with the series of prepared buffers we estimated *K* of DNA hybridization at each [Na^+^]^0^ (see violet points in Figure 4a). Taking into consideration that DNA strands are at 5 nM concentration, great excess of ions in environment (millimolar concentration scale, 6 orders of magnitude difference; thus, [Na^+^]^0^ ≫ [DNA]^0^), we can assume that the initial concentration of sodium ions is almost equal to the concentration at equilibrium [Na^+^]^0^ ≈ [Na^+^]^eq^. Therefore, we could estimate the relation *K* = *X*·[Na^+^]^*Y*^, where X = 9.62 × 10^12^ and *Y* = 2.51 (violet dashed line). Next, in a separate series of experiments, we prepared constant concentration of crowders at different concentrations of Na^+^ and again measured *K* (see green points in Figure 4a). The obtained *K* values were transformed to the sodium concentration using the previous relation (*K* = *X*·[Na^+^]^*Y*^). At a given concentration of buffer, the difference between calculated concentration of sodium ions without and with the presence of crowders allows us to calculate how many sodium ions got complexed by crowder molecule, [Na·CW]^eq^. Knowing this value, [Na^+^]^0^, and [CW]^0^ using eq 2 we could estimate κ. This methodology was repeated for a series of multiple buffer concentrations and various crowders at 40 wt %.

**Figure 4:**
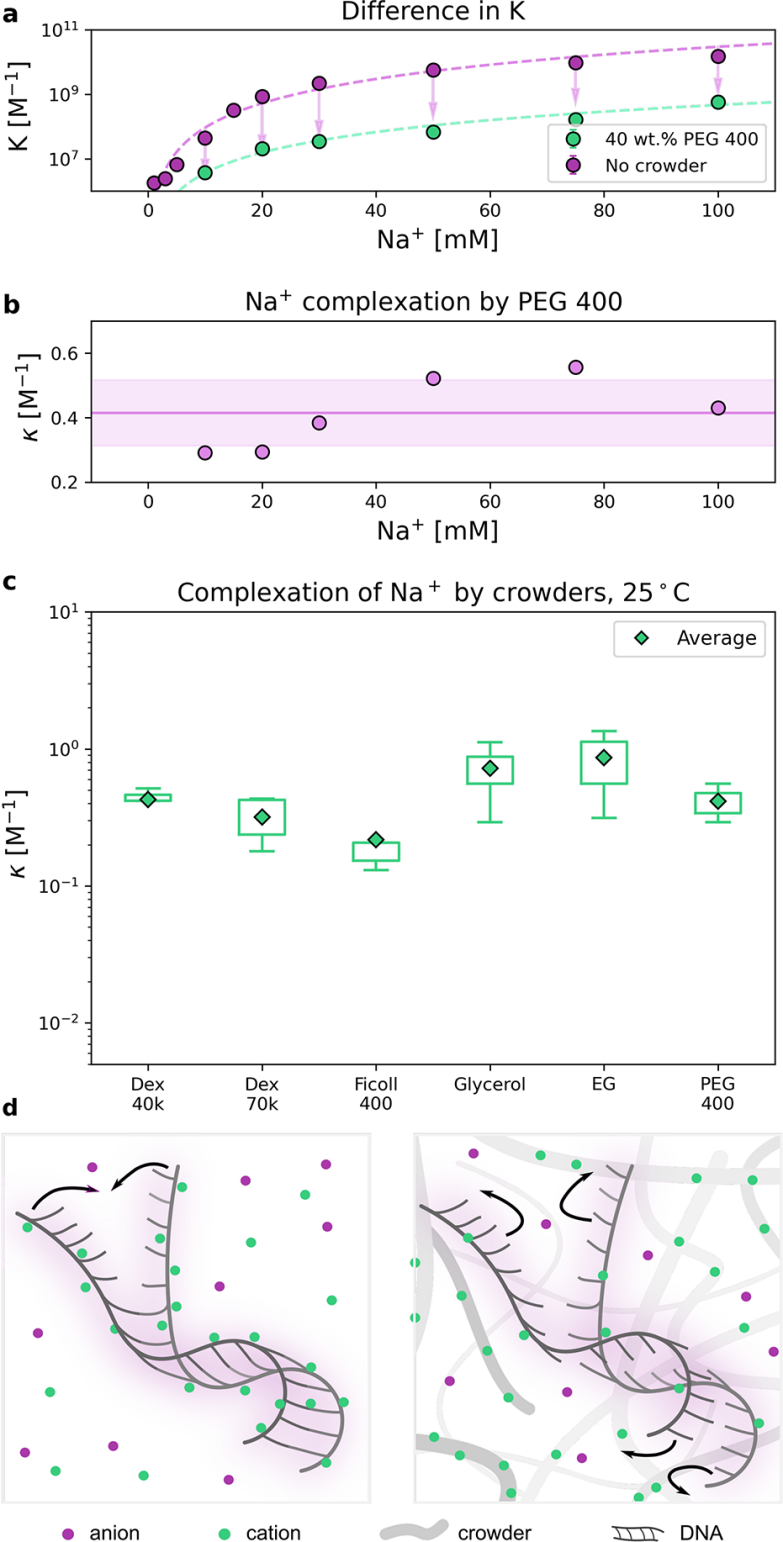
(a) Hybridization constant depending on the concentration of sodium ions without the addition of cosolute (violet dots) and in the presence of 40 wt % PEG 400 (green dots). The arrows in the plot correspond to the difference between the *K* values and thus the number of complexed sodium cations. (b) The sodium ion complexation by PEG 400 at various concentrations of sodium cations in the solution. The violet line represents the average value of the constant κ determined per one PEG 400 monomer (*n*_mer_ ≈ 8.5). Considering those results, we infer that the complexation of sodium ions by crowders, but not its direct interactions, is in fact mostly responsible for the changes in equilibrium constants. (c) Comparison of determining sodium complexation equilibrium constant by different crowders, excluding depletion force interaction. (d) (left panel) In an ionic solution, the partially charged compound is surrounded by an electrical double layer. Sodium cations screen negative charge of the DNA backbone, which facilities the complex formation. (right panel) After the addition of crowders, sodium cations are getting complexed by weak interaction with crowder molecules. In high concentration of crowders, repulsion of the DNA strands became more pronounced because of reduction of screening charges on the DNA backbone.

In the example experiment with PEG 400, using the presented methodology we determined ion complexation equilibrium constant, κ (see Figure 4b), and averaged it over a series of data points. The calculated complexation constant estimated with brightness analysis is 0.41 ± 0.10 M^–1^. We measured cosolutes differentiated in molecular sizes and chemical structure (only oxygen was used as a heteroatom in functional groups). The difference in size is not pronounced, especially in the case of crowders similar in structure (sugar moiety), *e.g.*, big dextran of average molecular weight 70 kDa and ficoll, ∼400 kDa. The determined κ values calculated per crowder molecule or monomer (in the case of polymers) are summarized in Figure 4c. In section S6 of the Supporting Information we discuss how addition of crowders can change other properties of the solution, such as dielectric constant, viscosity, pH, or activity.

All the collected data and calculations together with the proof that adding a certain amount of ions reverse the *K* constant to the higher value suggest complexation of sodium ions by crowders. We also tried to prove it directly by potentiometric measurements using ion-selective electrodes (ISEs) in the crowded solutions as well as with the crowders separated by the dialysis membrane. However, we met experimental difficulties, which are described in detail in the Supporting Information, section S7.

The influence of crowders is related not only to the effect on IS but also to the direct impact on the substrates of the reaction. For instance, depending on the type (*e.g.*, ionic or nonionic) and concentration of crowders, the enzymatic activity can decrease or increase, and protein stability is altered.^44^ It was shown that the presence of the crowder molecule near the local neighborhood of the protein substrate may affect its dielectric properties and its hydration structure.^45^ Crowders bind water molecules, which can influence the effective amount of available solvent.^46^ In section S8 of the Supporting Information, we show the estimated effect of water-binding by crowder molecules and how it may affect concentrations of ions and thus the κ constant. Additionally, the effect of depletion forces that occurs when crowders exclude the effective reactive volume should also be considered and included in the calculation (see Figure 5).

**Figure 5:**
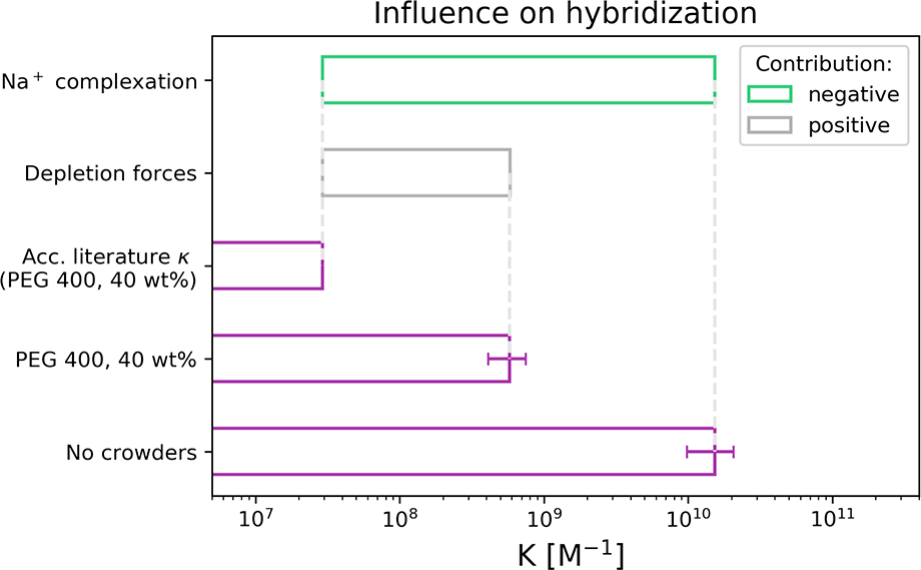
Contribution of interactions in a crowder system. The bar plots (violet) represent experimentally measured *K* values of 13 bp oligonucleotide hybridization in 100 mM buffer: without crowders, with presence of 40 wt % PEG 400, and in theoretical system where 73 mM of sodium ions were complexed by 40 wt % PEG 400 (κ = 1 M^–1^) without secondary interactions. The future quantitative analysis forces separation of the contributions of ion complexation and the depletion forces.

To conclude this study, we applied the brightness analysis method to investigate two factors that affect noncovalent complex formation: crowded environment and ionic strength. We showed that an increase in interactions between substrates is increased by ionic strength. However, after exceeding a certain value, it augmented the reducing effect on the bound fraction, which is unintuitive at first sight. This observation may be especially important for further analysis of biochemical reactions of organisms in a highly saline environment or with less access to water. In further analysis, we showed the applicability of the hybridization of DNA reaction as an indicator of sodium ion concentration. The experiments performed in the crowded environment showed that ion complexation, but not molecular crowding, may be responsible for the most changes in *K* values of biochemical interactions. In addition, we confirmed the results obtained by measurements using an ion-selective electrode. On the basis of this observation, we determined the complexation of sodium cations (on average, κ ≈ 0.49 M^–1^) by the most popular crowders differentiated in size and chemical structure. Our results will help to plan precise *in vitro* experiments to mimic conditions in living cells.

## Supporting Information Available

Detailed description of materials and experimental methods, microscope setup, the full description with equations of brightness-based method to determine the equilibrium *K* constant of biochemical reactions, validation of *K* constant of DNA hybridization by FRET method, changes of solution properties (*e.g.*, viscosity and pH) after addition of crowders, results of measurements of sodium cation complexation by nonionic crowders with the use of ion-selective electrode, and the discussion and results of water complexation by nonionic crowders (PDF)
