# Supplementary material for: Ion Complexation Explains Orders of Magnitude Changes in the Equilibrium Constant of Biochemical Reactions in Buffers Crowded by Nonionic Compounds
Source: J Phys Chem Lett. 2021 Dec 28;13(1):112–7. doi: 10.1021/acs.jpclett.1c03596 (PMC8762655; doi:10.1021/acs.jpclett.1c03596)
Supplement: Supplementary file 1 — jz1c03596_si_001.pdf [file jz1c03596_si_001.pdf]

Supporting Information for:
Ion Complexation Explains Orders of Magnitude Changes in the Equilibrium Constant of Biochemical Reactions in Buffers Crowded by Non-ionic Compounds.

Krzysztof Bielec,[†,‡] Adam Kowalski,[†] Grzegorz Bubak,[†] Emilia Witkowska Nery,[†] Robert Holyst[†*]

†Institute of Physical Chemistry, Polish Academy of Sciences, Kasprzaka 44/52, 01-224 Warsaw, Poland
‡Institute of Chemical Sciences and Engineering EPFL CH C2 425, Bâtiment CH, Station 6 Lausanne CH-1015, Switzerland

* rholyst@ichf.edu.pl

1 Materials

As a buffer we only use Phosphate Buffer (PB, pH= 7.4) which was prepared in necessary molar concentration/ionic strength. For instance, 100 mM stock solution was prepared by diluting 19 mL of 0.2 M $NaH_2PO_4\cdot H_2O$ and 81 mL of 0.2 M $NaHPO_4\cdot 7H_2O$ with 100 mL MiliQ water with 0.002 % Tween 20 used as the surfactant.

Oligonucleotides: The experiments were conducted on a thirteen-base pair complementary DNA strands (5' ATC GTG TAG GCA T 3') purchased from IBA GmbH, Germany. The single strands' stock solution at concentration of 100 μM was prepared in standard TE buffer and stored at -20 °C. The designed structures prevent the formation of secondary structures. Oligonucleotides were bought in two configurations. The first pair was strand labeled with ATTO488 and unlabelled complementary strand, the second was double-labeled pair with ATTO488 and ATTO647N at the same end after hybridization.

Cosolutes such as dextran 40k and dextran 70k (biosynthesized by the nonpathogenic organism *Leuconostoc*), Ficoll 400 and PEG 400 were purchased from MilliporeSigma, Germany. Both ethylene glycol and glycerol 99.5 wt.% were bought from Chempur, Poland.

2 Microscope setup

We performed fluorescence measurements on a Nikon C1 inverted confocal microscope upgraded with PicoQuant LSM module and PicoHarp 300 Time-Correlated Single-Photon Counting setup (TCSPC). We mounted Nikon Plan Apo 60x (NA=1.2) water immersion objective. As an excitation source, we used a pulsed (485 nm) diode laser (PicoQuant GmbH, Germany) with a frequency of 40 MHz. We optimize the laser power used in the experiments to obtain the highest possible signal to noise ratio that prevents photodamaging of the labeling dyes. The power of the laser (power meter PM 100, Thorlabs) was set on the average at 50 ± 5 μW. The measurements were undertaken within a climate chamber (OkoLab, Italy) at 25 ± 0.5 ℃. We loaded the samples into a glass-bottom container (ibidi GmbH, Germany) and positioned the focal volume at a distance of 10 μm from the edge of the glass surface. We collected the fluorescence signal filtered by 488 long-pass filter by two Single Photon Avalanche Diodes (MPD and PerkinElmer). The focal volume size was estimated by calibration using Rhodamine 110 (Sigma-Aldrich). For single sample point at given conditions (i.e., substrates ratio concentrations, ionic strength, crowders concentrations) photons during an experiment were collected for periods 90-120 seconds to prevent photo-bleaching. The setup was controlled by the PicoQuant Sepia II laser controller together with SymphoTime 64 software. Further data analysis was performed by self-written Python scripts.

3 Brightness method

We determine the equilibrium constant (K) in given experimental conditions by the brightness analysis method.[1] Molecular brightness (MB) of a fluorescent molecule is defined as the number of photons, N_{photons} emitted by a molecule in a time of period, t. It depends on the molecular properties of the chromophore such as quantum yield, excitation conditions (e.g., wavelength, laser power) and experimental setup (e.g., detector efficiency, emission filters). MB is sensitive to any local environment fluctuation (e.g. change of pH, solvent or formation of complex).[2–4] Under a given experimental condition, MB is expressed as:

$$\frac{N_{photons}}{t \cdot N_{molecules}} = MB \tag{1}$$

The emitted photon signal (χ_0) is proportional to the concentration C_A of the fluorophore and its intrinsic MB (α) excited inside the focal volume V_0. This can be written as:

$$V_0 \cdot \alpha \cdot C_A = \frac{N_{photons}}{t} = \chi_0 \tag{2}$$

The equilibrium constant of DNA hybridization between labeled oligonucleotide strand (A) and non-fluorescent complementary strand (B) is given by:

$$\mathrm{A} + \mathrm{B} \xrightleftharpoons{K} \mathrm{AB} \quad , \quad K = \frac{C_{AB}^{eq}}{C_A^{eq} \cdot C_B^{eq}}$$

After hybridization formed complex AB posses MB γ different than α. Thus the increased number of fluorescent components changes Equation (2) to:

$$V_0 \cdot \left(\alpha \cdot C_A^{eq} + \gamma \cdot C_{AB}^{eq}\right) = \chi_1 \tag{3}$$

After transformation it can be rewritten to its final form:

$$V_0 \cdot \alpha \cdot \left[C_A - C_{AB}^{eq}\right] \cdot \left[1 + \frac{\gamma}{\alpha} \cdot K \cdot \left(C_B - C_{AB}^{eq}\right)\right] = \chi_1 \tag{4}$$

Here C_{AB}^{eq} can be analytically solved to determine the equilibrium concentration of complex as the function of three experimentally known variables $C_{AB}^{eq} = f\left(C_A, C_B, K\right)$.

In our previous works, we used the brightness equation to determine equilibrium constants of oligonucleotide pairs in different labeling systems, as well for the determination of reaction mechanism for drug-DNA interaction.[1, 5]

The final form of Equation (4) is solved with previously determined parameters: 1) the confocal detection volume V_0. We defined it during the calibration of the experimental setup; 2) MB of single fluorescent strand α together with initial concentration C_A are determined in FCS experiment; 3) The MB of the complex is measured in experiment where second substrate B is in excess over A. Equation (4) is fit to the experimental series where the concentration of fluorescent component is set constant $C_A = const$ and concentration of second substrate (C_B) is varied. In this study, the concentration of fluorescent substrate (13 nucleotides ssDNA ATTO488 labeled, for details see page S2 – Materials) was kept constant at 5 nM, and the concentration of nonfluorescent substrate (complementary strand) varied. As the ratio $\frac{C_B}{C_A}$ increases, χ as a function of C_B begins to resemble binding isotherm; see Figure S1.

The brightness analysis model function (Equation 4) was fitted with the least square method using Python's lmfit library (solution for C_{AB} is provided elsewhere).[1, 6] As a result we obtain the values of variables (i.e., equilibrium constant, K and concentration of fluorescent component C_A) with corresponding uncertainties of estimation provided as standard errors. The inflection of the change in photons count rate, χ_1 characterizes the strength of interaction between molecules (the bigger inflection the bigger K, see Figure S1).

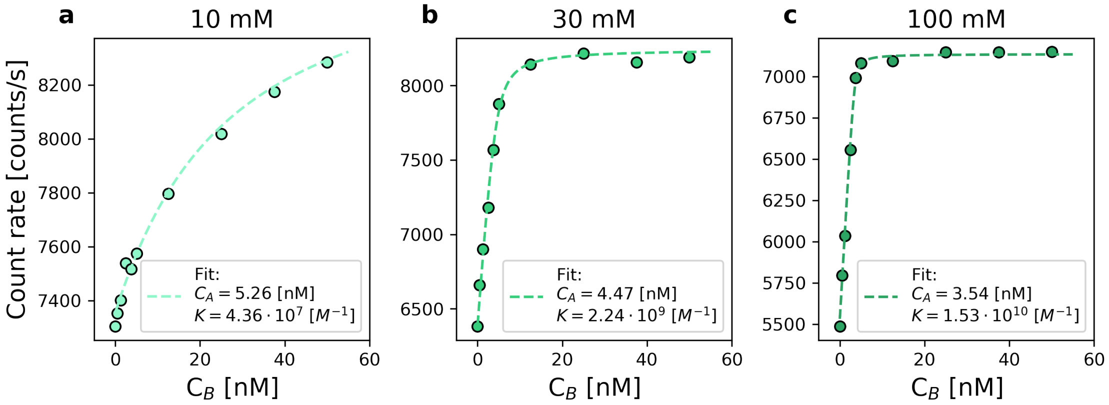

Fig S1: Change in count rate during product formation in relation to the change in nonfluorescent strand concentration. The time required to establish equilibrium is crucial as on single nanomolar scale it may last several hours.[7] Therefore, all experimental series were measured at equilibrium after 24 hours incubation. Plots for different concentrations of phosphate buffers: **a** 10 mM, **b** 30 mM, **c** 100 mM.

4 Validation by FRET pair

In our previous work, we have shown that the K determined by brightness method are same as the values obtained with the FRET.[1] Although, we investigated influence of ionic strength on the hybridization K constant of double labled pair to ensure obtained results. We measured K using FRET double-labeled complementary oligonucleotides with donor and acceptor dyes on the same end (ATTO 488 as donor and ATTO 647N as acceptor) of the formed complex, see Figure S2.

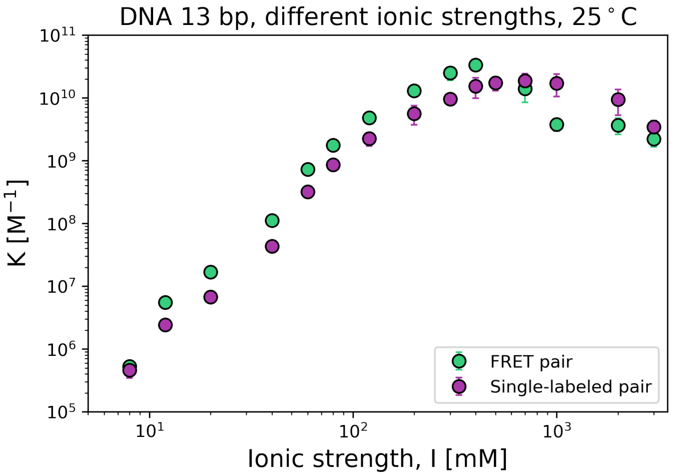

Fig S2: The influence of ionic strength on hybridization of FRET double labeled pair (green points) and single labeled pair (violet points) measured by brightness analysis.

The K value of double labeled pair (used for FRET analysis) hybridization is three times higher than constant obtained for single labeled pair (used for brightness analysis). We shown that this additional attraction is caused by the π-π stacking between dyes in double labeled pair.[1] This relationship persists up to 400 mM of ionic strength, then the trend is reversed. The highly saline system most probably affects the π-π stacking between ATTO dyes' aromatic groups.

5 Effect of crowded environment at various ionic strengths

We investigated complexation of most popular crowding agents used in biochemical experiments: dextran, ficol, polyethylene glycol and glycerol. The differences in K compared to the function obtained in non-crowded environment are presented in Figure S3.

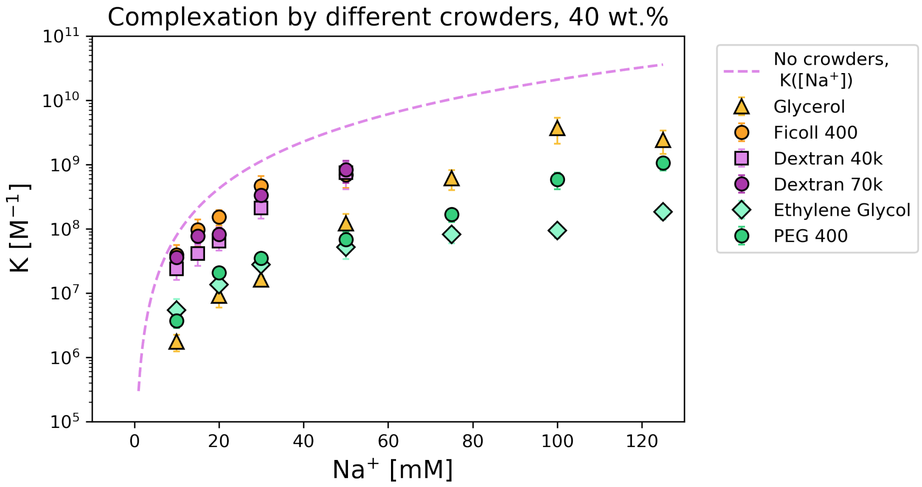

Fig S3: The results obtained for all the crowding agents considered in this work.

6 Change of solution properties after addition of crowders

The ion complexation hypothesis was taken into account, paying attention to the possibility that after addition of crowders other properties of the solution might be changed, such as viscosity, dielectric constant, pH or activity.

The change of dielectric constant can be calculated after assumption that both water and crowder molecules are ideally mixed. Using Oster's rule the dielectric constant of mixture can be calculated by determination of polarization. There, polarization of fluid mixture can be calculated as: where, pm is the polarization per unit volume of mixture, n is number of components, xi vi and pi are mole fraction, molar volume and polarization of component i, respectively. In literature dielectric constant changes from 78.36 in pure water to 56.74 for PEG 400 40 wt.%, which is 28 % change in total.[8]

We measured diffusion coefficients of the studied DNA (both in single-stranded and double-stranded form) in all crowding systems which is a measure of viscosity sensed by nucleic acids. The diffusion coefficient was determined for single-strand and double-strand DNA in the presence of the studied crowders in various wt.%, see Figure S4. Measurements were performed using the Fluorescence Correlation Spectroscopy, FCS. As crowder concentration increases, the crowder molecules constrains the diffusion of DNA substrates (diffusion coefficient decreases) due to the excluded volume. One base pair of DNA corresponds to approximately 0.34 nm of length along the strand.[9] Thus, studied oligonucleotides have a length of 4.42 nm. The hydrodynamic radius determined by FCS measurements is 1.6 nm for ssDNA and 1.7 nm for dsDNA. The differences in the theoretical length of DNA and its hydrodynamic radius result from the flexibility of oligonucleotides.

In comparison to buffer without coslutes, addition of high concentration of crowders (40 wt.%) does not change significantly pH in the reaction system, see Figure S5a. Among all used cosolutes in experiments only in ethylene glycol and PEG 400 solutions a slight increase in pH was observed. The DNA is chemically stable in pH regime between 5 to 9, thus we did not expect big influence on association and dissociation of DNA duplex.[10, 11] To ensure, we checked whether such change of pH may influence K of DNA hybridization in the regime from 6.5 to 8, see Figure S5b. The value of pH did not decrease value of K, even a slight increase was observed.

The activity is a matter of dispute. In highly crowded environment the diffusion of reactants is limited. Substrates probe the system much slower than in non-crowded environment, thus association constant k+ decreases. Once complex molecule is formed substrates the reactivity volume is reduced, thus more acts of collision can occur (k- decreases). Those two effects partially cancel each other out when equilibrium constant is calculated K= k_+/k_-. In our previous work we described that act of multiple rebindings at low concentrations of substrates is normalized by all acts in the system.[7, 12] Thus, we suspect that the equilibrium constant will not depend on viscosity. Although, the time required to establish equilibrium is crucial as on single nanomolar scale it may last several hours. Therefore, all experimental series were

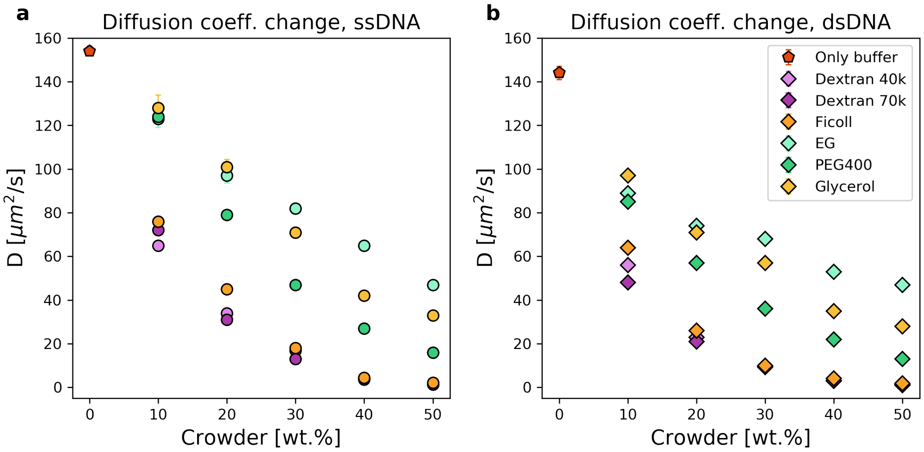

Fig S4: The influence of crowded environment on diffusion coefficient of **a** single-strand DNA and **b** double-strand DNA measured by FCS. Before experiment samples were incubated in crowded environment over night to establish equilibrium.

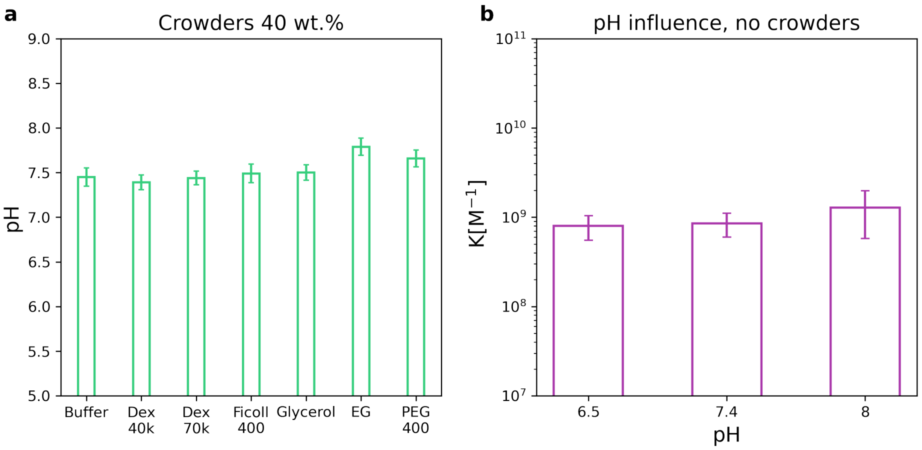

Fig S5: **a** Change in the pH of phosphate buffer in the presence of 40 wt.% of crowders. **b** Constant K in 20 mM PB buffer for various pH.

measured at equilibrium after 24 hours incubation.

7 Ion-selective measurements

We used the potentiometric method to confirm ion complexation by crowders. Experiments presented in Figures S6 and S7 required ∼ 1 mM sensitivity of sodium ions detection. Therefore, to observe sodium complexation, we used an ion-selective electrode (perfectION, Mettler Toledo) and calibrated it in the range from 50 μM to 200 mM of phosphate buffer pH 7.4 at 25 °C, see Figure S6. Sodium-ion activity was calculated with the Truesdell-Jones model.

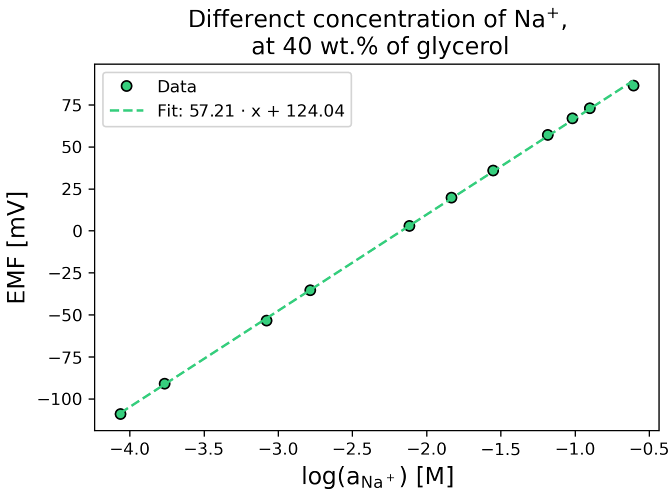

Fig S6: Calibration curve for different concentrations of phosphate buffer at pH 7.4, T = 25 °C.

Next, we checked whether non-ionic crowders in water change the potential of the electrode, see Figure S7a. There is no relationship between concentration and electrode potential for glycerol and ethylene glycol. Yet, for the remaining crowders, the potential rises with the increase in crowder concentration. This variation is probably due to electrode fouling by polymers or contaminants. We have first conducted measurements with standard laboratory made ion-selective electrodes equipped with a plasticized polyvinyl chloride membrane. Those electrodes presented expected slope and detection limit in standard aqueous solution but did not respond to changes in sodium concentration when the measurement was performed in high PEG concentration. This result suggests fouling of the electrode surface may take place in such crowded solutions.

Finally, we measured the change in sodium ion potential in the presence of crowders, see Figure S7b. We kept the concentration of crowders constant at 40 wt.% and varied the concentration of PB buffer pH 7.4 at 25 °C (few micromolar to millimolar). We prepared sample series 24 hours prior to the experimental session to establish the equilibrium of the interactions. Initially electrode potential did not change until a particular crowder concentration was added. This result suggests ion complexation by crowders. However, the inflection point does not reach expected crowder concentration values. Knowing from brightness analysis, for example, 40 wt.% glycerol would complex ∼100 mM of sodium ions, yet one can see from Figure S7b that electromotive force (EMF) for glycerol begins to vary at 0.2 mM of sodium ion concentration.

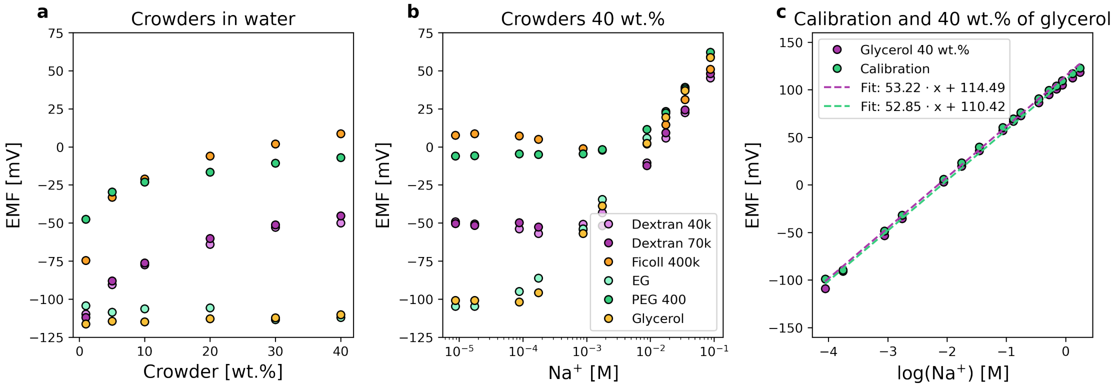

Fig S7: Potentiometric measurements of **a)** crowders in water **b)** PB buffer different concentrations pH 7.4 at constant 40 wt.% crowder concentration, and **c)** PB buffer different concentrations pH 7.4 in water (calibration) and at constant 40 wt.% glycerol concentration (larger concentration regime), T = 25 ℃.

Table S1: Analytical parameters of the ion selective electrode calibration curves obtained with different crowders.

**Crowder (40 wt.%)**	**Linear range** $\mathbf{c_{Na^+}}$ **[M]**	**Sensitivity** $\mathbf{[mV/log(c_{Na^+})]}$
None	$8.7{\cdot}10^{-5}$– 1.320	52.85
EG	$8.7{\cdot}10^{-5}$– 0.087	53.28
Glycerol	$8.7{\cdot}10^{-5}$– 1.320	53.38
PEG 400	$8.7{\cdot}10^{-3}$– 0.087	51.28
Dextran 40k	$8.7{\cdot}10^{-4}$– 0.087	48.58
Dextran 70k	$8.7{\cdot}10^{-4}$– 0.087	53.57
Ficoll 400k	$8.7{\cdot}10^{-3}$– 0.087	49.78

Table S1 resumes the analytical parameters of the calibration curves obtained with different crowders. Values for pure buffer are added for reference. Sensitivity is stated as the change in EMF per one unit of $\log(C_{Na+})$. We did not find a proper model to calculate activity coefficients in the presence of crowders, for this reason sensitivity is based on concentration and not the actual activity of sodium in solution. To calculate the activity coefficient in case of the measurement without crowders we used Truesdell-Jones model, which is applicable also for solutions of high ionic-strength. In this case the slope of the calibration curve is 57.21 mV/ $\log(a_{Na+})$, which is close to theoretical (59.16) for single charged ions, such as sodium.

Type of crowder does not have a high impact on the sensitivity of the assay, as values oscillate around 50 mV/$\log(C_{Na+})$ (probably would be slightly higher if calculated for activity). The lower limit of the linear range of the sensor is highly influenced by the crowder type. Addition of ethylene glycol and glycerol had no effect on the linear range as compared with the pure buffer. For both types of dextran lower limit shifted from $8.7 \cdot 10^{-5}$ to $8.7 \cdot 10^{-4}$ and for PEG 400 and Ficoll 400k even to $8.7 \cdot 10^{-3}$. The upper linear limit was not affected and extended at least till 1.3 M for both pure buffer and 40 wt.% glycerol (Fig. S7c). In Figure S7a it is shown that the change of concentration of glycerol from 1 to 40 wt.% results in the EMF change of 6mV. Similar difference around 6 mV is observable for the calibration curve performed in pure buffer and in buffer with 40 wt.% glycerol (Fig. S7c).

8 Influence of crowders on DNA hybridization equilibrium constant

The experimental series here are the same as shown in Figure 2 in the main manuscript, however with recalculated X axis to monomer concentration of given crowder in mol/dm^3.

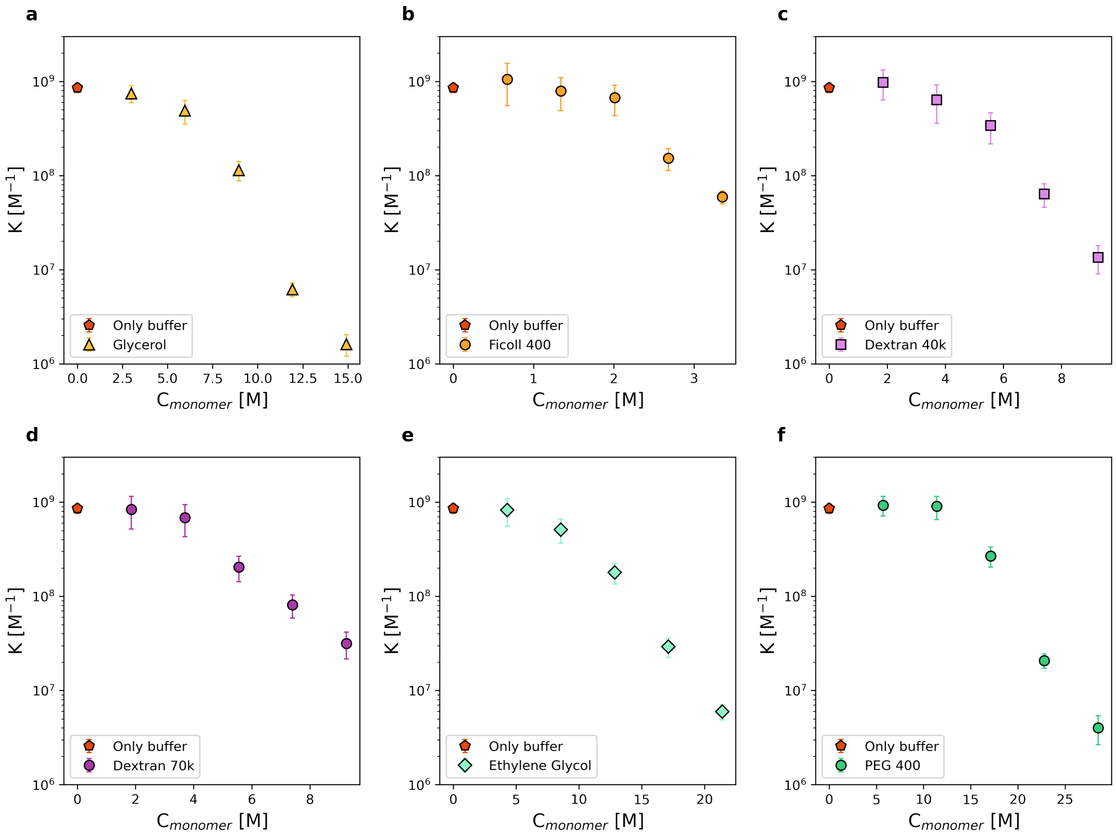

Fig S8: The hybridization reaction was measured at constant ionic strength in the presence of various crowder agents in concentrations from 0 to 50 wt.%: **a)** glycerol, **b)** ficoll 400, **c)** dextran 40k, **d)** dextran 70k, **e)** ethylene glycol, **f)** PEG 400.

9 Water complexation

Since crowders bind water molecules, the amount of solvent decreases, and sodium ion concentration increases, see Figure S9a. Here we consider this effect and calculate corrected κ values over bound water by PEG 400, according to Branca, C., et al.[13]

The effect of the change of ion concentration in the solution was determined based on the values given for Figure S9a. There, 40 wt.% concentration of PEG 400 increase sodium ions concentration by two folds. Knowing this, we recalculated effective concentration of $[Na^+]^0$ and $[Na \cdot CW]^{eq}$ (see Equation 5 in main text) simply just by multiplying reference concentrations of data points of K measured in the presence of 40% wt. crowders by factor of 2.08. After this, we once again performed calculation steps described in main text and obtained corrected values of κ, see Figure S9b.

The given corrected values are estimated and determined with respect to ion complexation by PEG 400. For each crowder, the effect of the associated water molecules should be determined separately and included in the calculations.

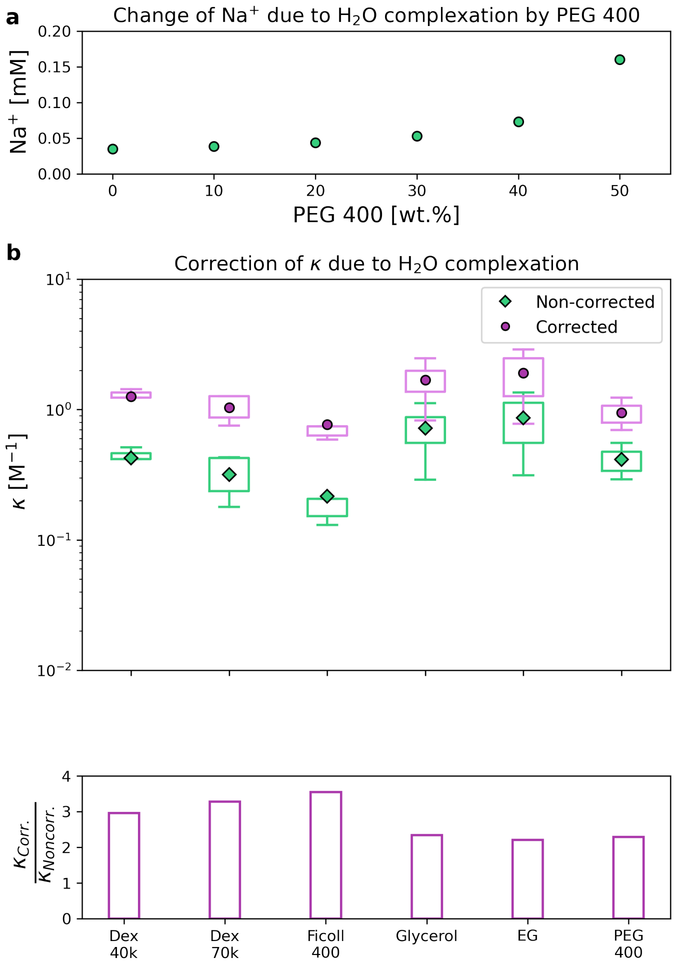

Fig S9: Ion complexation by different crowders corrected by the exclusion of water molecules from the volume of the reaction solution.

References

(1) Bielec, K.; Bubak, G.; Kalwarczyk, T.; Holyst, R. *Journal of Physical Chemistry B* **2020**, *124*, 1941–1948.

(2) Rachofsky, E. L.; Osman, R.; Ross, J. A. *Biochemistry* **2001**, *40*, 946–956.

(3) Dos Remedios, C. G.; Moens, P. D. *Journal of structural biology* **1995**, *115*, 175–185.

(4) Marras, S. A.; Kramer, F. R.; Tyagi, S. *Nucleic acids research* **2002**, *30*, e122–e122.

(5) Zhou, Y.; Bielec, K.; Pasitsuparoad, P.; Hołyst, R. *Analyst* **2020**, *145*, 6600–6606.

(6) Newville, M.; Stensitzki, T.; Allen, D.; Ingargiola, A. LMFIT: Non-Linear Least-Square Minimization and Curve-Fitting for Python, doi: 10.5281/zenodo. 11813, 2014.

(7) Bielec, K.; Sozanski, K.; Seynen, M.; Dziekan, Z.; ten Wolde, P. R.; Holyst, R. *Physical Chemistry Chemical Physics* **2019**, *21*, 10798–10807.

(8) Panraksa, P.; Tipduangta, P.; Jantanasakulwong, K.; Jantrawut, P. *Membranes* **2020**, *10*, 376.

(9) Damaschun, G.; Damaschun, H.; Misselwitz, R.; Pospelov, V.; Zalenskaya, I.; Zirwer, D.; Müller, J.; Vorobev, V. *Biomedica biochimica acta* **1983**, *42*, 697–703.

(10) Roberts, R. W.; Crothers, D. M. *Science* **1992**, *258*, 1463–1466.

(11) Zhang, J.; Lang, H. P.; Yoshikawa, G.; Gerber, C. *Langmuir* **2012**, *28*, 6494–6501.

(12) Kalwarczyk, T.; Bielec, K.; Burdzy, K.; Holyst, R. *Physical Chemistry Chemical Physics* **2021**, *23*, 19343–19351.

(13) Branca, C.; Magazu, S.; Maisano, G.; Migliardo, F.; Migliardo, P.; Romeo, G. *The Journal of Physical Chemistry B* **2002**, *106*, 10272–10276.
